# Evaluation of weaning strategies using an automated milk replacer feeder on growth performance and physiological responses in Hanwoo calves

**DOI:** 10.5713/ab.260116

**Published:** 2026-06-01

**Authors:** Hyerin Nam, Seongjin Kim, Seo-Ho Oh, Danil Kim, Younghye Ro, Sang-Kee Kang

**Affiliations:** 1 Graduate School of International Agricultural Technology, Seoul National University, Pyeongchang, Korea; 2 Asia Pacific Ruminant Institute, Icheon, Korea; 3 Institute of Green-Bio Science & Technology, Seoul National University, Pyeongchang, Korea; 4 College of Veterinary Medicine and Research Institute for Veterinary Science, Seoul National University, Seoul, Korea; 5 College of Veterinary Medicine, Kangwon National University, Chuncheon, Korea; 6 Research Institute of Agriculture and Life Sciences, Seoul National University, Seoul, Korea

**Keywords:** Calf Nutrition, Feeding Management, Korean Native Cattle, Solid Feed Adaptation, Step-down Weaning

## Abstract

**Objective:**

To assess automated feeder milk replacer (MR) weaning strategies for Hanwoo (Korean native cattle) calves and identify a cost-effective program that improves growth, feed efficiency, and timely adaptation to solid feed.

**Methods:**

Four 60-d MR programs were compared with a standard control by changing one key factor: MR feeding frequency (T1; 4 vs. 6 meals/d), MR solids concentration (T2; 13% vs. 15%), MR duration in the early feeding period (T3; 30 d vs. 37 d), or the MR tapering strategy in the late feeding period (T4; linear vs. step-down). Performance outcomes included dry matter intake (DMI), average daily gain (ADG), feed conversion ratio (FCR), and weaning body weight, and physiological responses were assessed with complete blood counts (CBCs) and serum biochemical profiles (SBPs).

**Results:**

Across treatments, performance differences were driven primarily by the MR-to-solid-feed transition method and timing rather than total DMI. In T1, MR DMI tended to be lower, and ADG was the lowest (p<0.001), which is consistent with reduced appetite under frequent MR meals. T2 increased MR DMI but yielded only moderate growth, with higher diarrhea incidence and duration and higher aspartate aminotransferase and lactate dehydrogenase, suggesting greater metabolic load at 15% vs. 13% MR solids. Compared with the control, T3 and T4 improved ADG and FCR, with significant treatment effects (p<0.001), and increased weaning body weight (p = 0.001). T3 prolonged MR-centered feeding early, whereas T4 used step-down tapering to increase solid feed intake late and promote earlier solid feed reliance. CBC and SBP showed no added disturbance in T3 or T4.

**Conclusion:**

Both T3 and T4 increased growth and efficiency. T4 performed comparably with reduced MR and earlier solid feed dependency, indicating practical and economic benefits.

## INTRODUCTION

Calves, as young ruminants, rely primarily on milk during the early postnatal period until the rumen develops sufficiently to support substantial solid feed (SF) utilization [1,2]. In cattle, the nursing period is also important for acquiring passive immunity and supporting rapid body growth, while the gradual transition from milk to SF is essential for rumen development and successful weaning [3]. Once the digestive system has developed sufficiently to allow voluntary intake of adequate amounts of SF, a scientific and systematic weaning program is required to gradually reduce milk supply while progressively introducing SF sources such as concentrates and forages [4,5]. Nonetheless, the Hanwoo (native Korean beef cattle) industry in Korea has traditionally maintained a passive approach in which neonatal calves are allowed to suckle naturally from their dams for at least 3 months after birth [6]. However, Hanwoo cows produce about 4 L/day at peak lactation, substantially less than Holstein cows (25–30 L/day) [7]. Consequently, natural suckling may not consistently provide sufficient nutrients for optimal calf growth, thereby limiting the production of healthy and uniformly weaned feeder calves. In addition, suckling by the calf can delay postpartum estrus resumption, lower conception rates, and prolong calving intervals in dams, ultimately reducing reproductive and economic efficiency [8]. In contrast, the dairy industry has long used systematic artificial weaning programs based on milk replacers (MRs), in which calves are separated from dams at an early stage to standardize calf management and improve production efficiency [9]. Because calf growth efficiency and dam reproductive performance are key determinants of the competitiveness of Hanwoo farms, there has been increasing interest in adopting MR-based artificial weaning programs similar to those used in the dairy industry. Although MR products for Hanwoo calves have recently entered the market and been adopted by some farms, scientifically validated artificial weaning systems remain limited in Korea. In conventional manual MR feeding for neonatal calves, approximately 10% of the calf’s body weight in MR, prepared at an appropriate concentration, is commonly fed daily in two portions [10]. Considering the digestive physiology of young calves, multiple smaller feedings per day may provide a more even nutrient supply and reduce digestive burden [1]. However, feeding MR only twice daily for managerial convenience may result in insufficient intake, prolonged fasting, reduced growth rates, and metabolic diarrhea [11]. In addition, the need to calculate individual feeding volumes according to the birth date and growth stage of each calf increases the management burden.

To address these issues, automated MR feeding systems or robotic MR feeders have been developed [12,13]. These devices automatically dispense programmed amounts of MR according to each calf’s growth stage, improving management efficiency and calf productivity.

However, establishing an artificial MR feeding program for Hanwoo calves requires not only the introduction of automated feeding equipment but also the optimization of feeding structure during the pre-weaning and weaning periods. Feeding frequency, MR solids concentration, duration of the early MR-centered feeding period, and the pattern of MR reduction during the late feeding period may differentially affect nutrient intake, SF adaptation, growth performance, and physiological stability. Therefore, these program components need to be evaluated under Hanwoo calf-rearing conditions.

We hypothesized that the growth performance and physiological responses of Hanwoo calves would be influenced not only by the amount of MR supplied but also by the timing and structure of MR feeding during the transition from MR to SF.

Therefore, this study aimed to evaluate the effects of daily feeding frequency, MR solids concentration, early feeding period duration, and late-period MR reduction strategy on feed intake, growth performance, feed efficiency, and hematological and serum biochemical responses in neonatal Hanwoo calves managed using an automated MR feeder. The ultimate goal was to identify a practical MR feeding strategy suitable for Hanwoo calves under artificial rearing conditions.

## MATERIALS AND METHODS

### Management of animals

In this study, 32 newborn Hanwoo calves (12 females and 20 males) were subjected to artificial MR feeding for 60 d postnatally. All calves were separated from their dams immediately at birth, dried, weighed, and housed individually in wooden pens (length×width×height: 2.0 m×1.35 m×1.2 m) with 5 cm-deep dry sawdust bed. They were fed 2.4 L of commercial colostrum (Headstart; SSCL), a dried bovine colostrum powder, manually, prepared according to the manufacturer’s instructions (22.5% concentration in 45°C water), within 12 hours of birth. During the experimental period, the calves were fed a MR (Nukamel Yellow; Nukamel), a product formulated with whey powder, vegetable oils (coconut and palm oils), and vitamin and mineral supplements, using a monorail-type automatic MR feeder (CalfRail; Förster Technik) with different feeding methods for each test group, according to the experimental design. Briefly, the calf feeder system automatically reconstitutes the MR by mixing the powder with warm water to a programmed concentration, and the prepared milk is delivered to each pen by a dispensing unit running along a rail system. The automatic feeder was programmed with preset pen numbers, and the feeding allowance (volume and frequency) was regulated according to a preset feeding schedule. After each feeding, teats and dispensing lines were automatically rinsed to maintain hygiene. The feeding behaviors, including intake volume, and visit frequency, were recorded by the system. In addition to the MR, all experimental groups were provided *ad libitum* access to a calf starter (Golden Calf; DH Vital Feed), which was formulated mainly with cereal grains, plant protein sources, and vitamin-mineral supplements, and water in separate buckets. Once the calves were able to independently consume more than 300 g of calf starter per day, chopped oat forage (approximately 5 cm long) was mixed with the calf starter at a 9:1 weight ratio and offered as a SF. The SF and water were replaced daily, and the dry sawdust bedding was replaced every week. Calves exhibiting diarrhea were recorded along with duration of diarrhea days and orally administered a solution prepared by dissolving 100 g of electrolyte powder (Life-Aid; Norbrook) and 14 g of an antibiotic (Neodiaristin; Fatro) in 2 L of water twice daily. If diarrhea persisted for more than 4 d, the calves were injected with 2 mL each of GPS (Green Cross) and uniketoprofen (UNIBIOTECH), depending on their clinical condition. The nutritional composition of the experimental feed is shown in Table 1.

### Experimental design and feeding regimens

**Figure 1:**
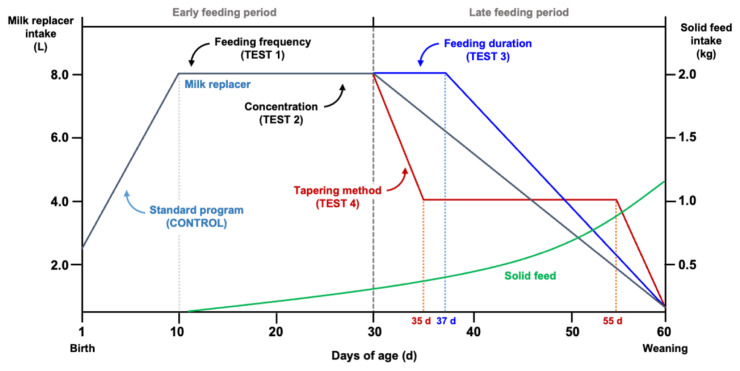
Schematic illustration of the 60-d milk replacer feeding program for Hanwoo calves. The feeding program consisted of an early feeding period (1–30 d; 1–37 d in T3) and a late feeding period (31–60 d; 38–60 d in T3). Calves were hand-fed artificial colostrum on day 1 after birth, and milk replacer (MR) feeding commenced from day 2. During the early period, calves were mainly fed MR, whereas during the late period, the MR allowance was gradually reduced, and solid feed intake increased as the calves approached weaning. The standard regimen described in Table 2 was followed in the Control group. To distinguish the effect of each variable, only one condition was modified for each treatment: T1, MR feeding frequency (6 times/d); T2, MR concentration (15%); T3, early feeding duration (37 d); and T4, step-down decrease in MR allowance. The green curve indicates an idealized increase in solid feed intake as the MR supply declines.

Five experimental groups were established to investigate the effects of different MR feeding strategies in Hanwoo calves. Calves were assigned sequentially to treatment groups according to their order of birth because the MR feeding program was initiated immediately after birth. Sex was not used as an allocation criterion; therefore, the sex distribution among treatments reflected the sex of calves available at each allocation point. All MR feeding programs lasted 60 days and comprised an early and a late feeding period. The early period encompassed postnatal adaptation to MR feeding until the preset maximum allowance was reached and maintained, whereas the late period, as the weaning transient time, involved a gradual reduction in MR as calves increased SF intake, continuing until complete weaning. The total daily MR allowance was fixed at 8 L across all groups; however, each group underwent a specific modification to the feeding regimen. The manipulated variables included feeding frequency and meal size, MR solids concentration, the duration of the early feeding period, and the tapering method used during the late feeding period. In the Control group (n = 6; 2 females and 4 males), calves received 2.0 L of 13% MR four times daily at 4-h intervals (07:00–19:00) during the early feeding period, followed by a linear-decrease (LD) tapering method during the late feeding period, in which MR was reduced by 266 mL/day until weaning. In T1 (n = 6; 2 females and 4 males), feeding frequency was increased to six times daily at 3-h intervals (07:00–19:00), with 1.5 L per meal; because the preset maximum daily allowance was 8 L, MR provision was stopped once this limit was reached, and LD tapering was applied as in the control. In T2 (n = 6; 3 females and 3 males), MR solids concentration was increased to 15% relative to the control, while the other feeding conditions, including LD tapering (266 mL/day), matched those of the control. In T3 (n = 7; 4 females and 3 males), the early feeding period was extended to 37 d; accordingly, during the LD tapering phase, MR was reduced by 348 mL/day until weaning. In T4 (n = 7; 1 female and 6 males), a step-down (SD) tapering method was applied during the late feeding period. Specifically, MR was reduced by 800 mL/day for the first 5 d of the late feeding period, maintained at 4 L/day for the subsequent 20 d, and then reduced again by 800 mL/day during the final 5 d before weaning. In all test groups, conditions were identical to those of the control group except for the designated variables under investigation. The details of all treatment groups and their respective feeding regimens are summarized in Table 2 and illustrated in Figure 1.

### Data collection and analysis

All calves were weighed immediately after birth, and their body weights were recorded once per week during the suckling period. Body size parameters (body length, chest girth, abdominal girth, withers height, and hip height) were measured on each weighing day, but initiated at 1 week of age due to practical constraints immediately after parturition. Individual daily MR intake was automatically recorded using the Calf Rail system, whereas SF intake (a mixture of calf starter and oat forage) was determined daily by manually measuring the residual feed amount in each pen. Blood samples were collected from the jugular vein at 2, 30, and 60 d of age immediately before the first morning MR feeding (prior to 07:00 h). A total of 10 mL of blood was obtained from each calf and divided equally into two 5-mL tubes: an EDTA tube (BD Vacutainer) for complete blood count (CBC) analysis and a heparinized tube (BD Vacutainer) for serum biochemical profile (SBP) analysis. For CBC analysis, samples were immediately analyzed using an automated hematology analyzer (Hemavet 950; Drew Scientific). For SBP analysis, blood samples were centrifuged at 2,012×g for 15 min (1580R; LABOGENE), and the separated plasma was stored at −80°C until analysis using an automated biochemical analyzer (BS-400; Mindray). Detailed information on the CBC and SBP parameters is provided in Supplements 1, 2.

### Statistical analysis

Statistical analyses were performed using SAS OnDemand for Academics (SAS Institute). Prior to mixed-model analysis, the assumption of normality was assessed using the Shapiro–Wilk test. When departures from normality were detected, model adequacy was further evaluated by examining residual normality, quantile–quantile (Q–Q) plots, and histograms, and the influence of potential outliers was assessed using studentized residuals. To ensure a consistent basis for comparing treatment effects across variables, all variables were analyzed using the same general mixed-model framework with PROC GLIMMIX. The model included treatment, sex, and the treatment×sex interaction as fixed effects, with calf included as a random effect.

For variables measured repeatedly within the same calf, including MR and SF intake during the early and late periods, body weight, blood parameters, and body measurements, the repeated factor was defined as period, week, or day depending on the variable. In contrast, overall-period variables, including MR intake, SF intake, total dry matter intake (DMI), average daily gain (ADG), feed conversion ratio (FCR), and body measurement gains, were calculated for each calf as a single value and analyzed without a repeated-measures structure. When a significant treatment effect was detected (p<0.05), pairwise comparisons among least squares means were performed using the Tukey–Kramer adjustment for multiple comparisons. Results are presented as means±standard error of the mean (SEM), and statistical significance was declared at p<0.05.

## RESULTS AND DISCUSSION

In this study, the MR feeding program was structured into an early feeding period and a late feeding period to examine how modifications in MR delivery and the MR-to-SF transition shape intake patterns and growth responses. Using a control program as the baseline, each treatment group varied only one factor: feeding frequency and meal size (T1), MR solids concentration (T2), early feeding period duration (T3), or the tapering strategy from MR to SF (T4). Growth performance and physiological responses were then evaluated.

Group-level DMI, ADG, and FCR, summarized separately for the early and late feeding periods, together with body weight trajectories from birth to weaning, are presented in Supplement 3. Body size profiles are provided in Supplement 4.

**Figure 2:**
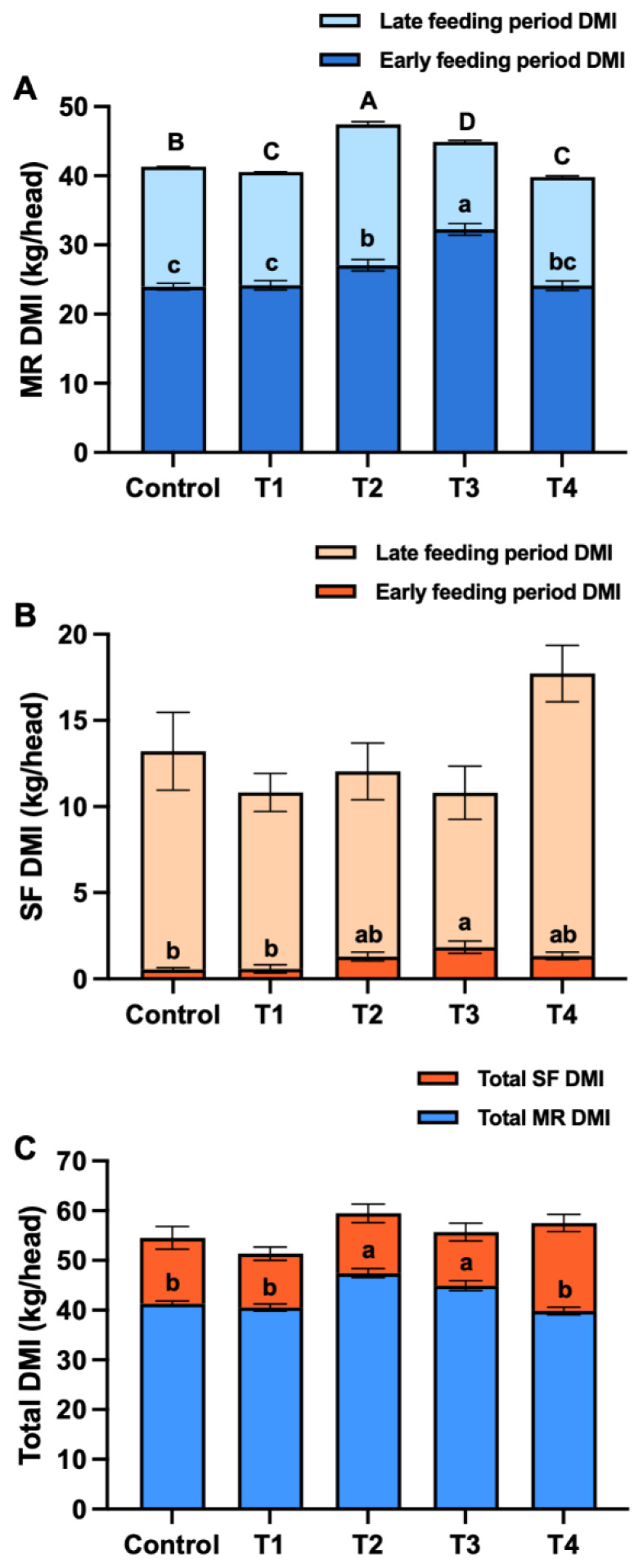
Cumulative MR DMI, SF DMI, and total DMI in early and late feeding periods during the MR feeding programs. (A) MR DMI during the early and late feeding periods. (B) SF DMI during the early and late feeding periods. (C) Total DMI of MR and SF over the entire experimental period. Bars represent mean±SEM. ^A–D, a–c^ Different letters indicate significant differences among treatments in the same sampling category (p<0.05). Groups sharing a letter are not significantly different. MR, milk replacer; DMI, dry matter intake; SF, solid feed; SEM, standard error of the mean; T1, feeding frequency and meal size; T2, MR solids concentration; T3, early feeding period duration; T4, tapering strategy from MR to SF.

**Figure 3:**
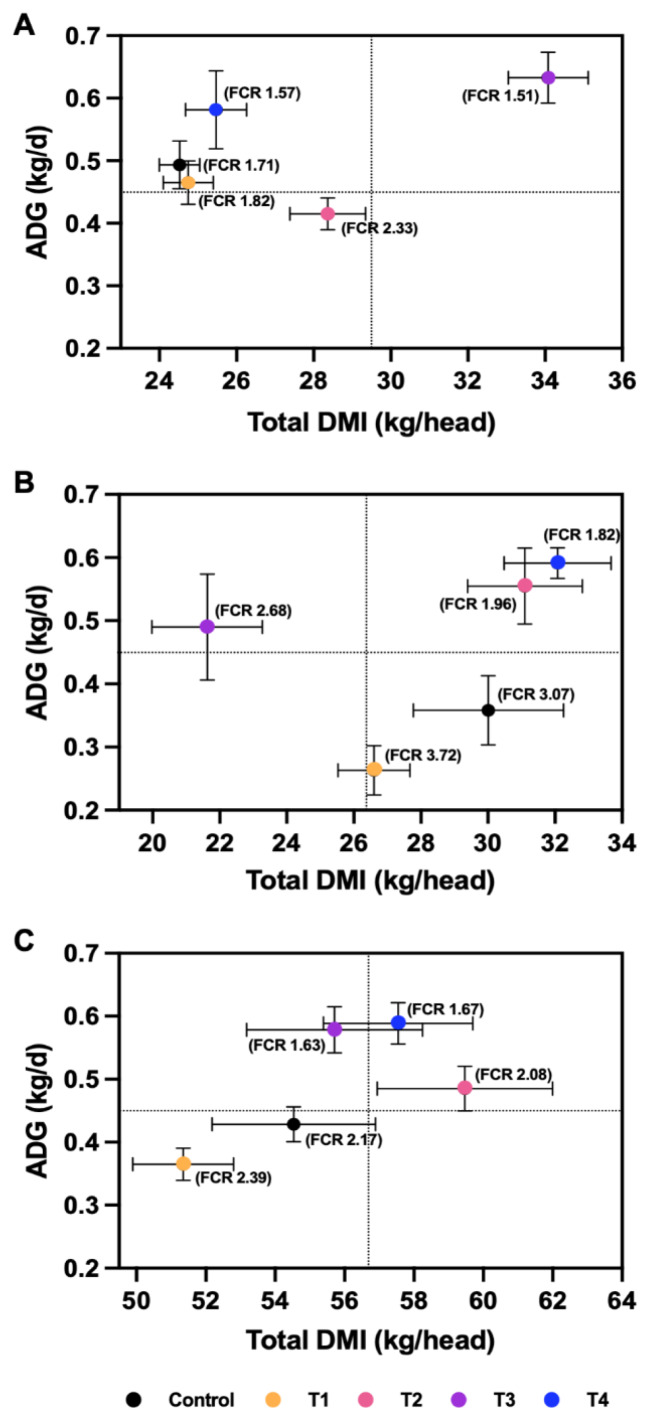
Performance maps illustrating the relationship among DMI, ADG, and FCR across feeding periods. The relationship among DMI, ADG, and FCR was illustrated in performance maps for the early feeding period (A), late feeding period (B), and total experimental period (C). Each point represents a treatment mean; horizontal and vertical bars indicate the standard error of the mean (SEM) for total DMI and ADG, respectively. FCR values for each group are displayed within each panel. ADG, average daily gain; FCR, feed conversion ratio; DMI, dry matter intake.

Considering that young mammals typically benefit from frequent, small-volume milk meals [14], the MR feeding schedule in T1 was modified from four meals per day (2.0 L/meal) to six meals per day (1.5 L/meal) (Table 2, Figure 1). However, calves in T1 frequently failed to consume the full allotment and occasionally skipped meals, resulting in one of the lowest cumulative MR intakes among the test groups (Figure 2A). SF (Calf starter:Oats = 9:1) intake also showed lower tendency than in the control (Figure 2B), leading to the numerically lowest total DMI (MR+SF) over the 60-d weaning period (Figure 2C). Consistent with these intake patterns, when nutrient supply depended primarily on MR, T1 exhibited low intake and low gain during the early feeding period (Figure 3A). In the late feeding period, during the transition from MR to SF, T1 shifted further downward along the ADG axis (Figure 3B). A plausible explanation is that frequent MR provision reduced hunger-driven motivation to consume SF, delaying the behavioral shift toward SF intake that underpins efficient growth during the weaning transition period. Consequently, when all periods were integrated, T1 clustered at the lowest DMI and ADG region of the performance map, indicating that the high-frequency regimen did not translate into improved growth (Figure 3C).

Given that Hanwoo milk generally has a higher total solids concentration than milk from Holstein and other dairy breeds [15], the MR solids concentration was increased from 13% to 15% in T2 (Table 2, Figure 1). A notable feature of T2 was that MR DMI during the late feeding period was highest among treatments, which contributed to a numerically higher total DMI (Figure 2A); however, this greater intake did not translate into more efficient growth. During the early feeding period, despite the higher MR solids concentration, cumulative MR DMI in T2 was not proportionally elevated (Figure 2A), and ADG and FCR were relatively poor on the performance map (Figure 3A). In the present study, diarrhea was observed in all groups at approximately 1 week after initiation of the feeding programs: 3 of 6 calves (mean duration, 4.0 d) in the control; 4 of 6 (3.5 d) in T1; 6 of 6 (4.8 d) in T2; 6 of 7 (2.7 d) in T3; and 5 of 7 (2.2 d) in T4 (data not shown). Notably, all calves in T2 exhibited diarrhea and the mean duration was the longest, which may help explain the inferior ADG and FCR during the early feeding period in this treatment. Neonatal diarrhea is common in calves fed MR and may arise from both infectious and nutritional causes, including increased osmolality with higher MR total solids [16]. Collectively, these results suggest that the higher-solids regimen in T2 imposed a greater transient digestive challenge, thereby contributing to the early-period depression in growth performance and efficiency relative to the other treatments. Although Hanwoo milk has a higher total solids concentration (approximately 15%) than Holstein milk (approximately 12.5%), milk yield during natural suckling in Hanwoo cows is typically only about 4 L/d even at peak lactation [7], which likely limits the overall solute (osmotic) load delivered to the gastro-intestinal tract. In the present study, because the maximum MR allowance was designed to reach 8 L/d, feeding a 15% MR in T2 may have increased the osmotic load relative to the 13% MR programs, thereby contributing to more frequent and prolonged diarrhea and to less efficient utilization of nutrients in the early feeding period. During the late feeding period, T2 maintained relatively high MR-derived DMI (Figure 2A), whereas SF DMI was broadly comparable among treatments and was marginally lower than in the control group (Figure 2B). Thus, the higher intake in this period appeared to be driven mainly by the higher-solids MR rather than by increased reliance on SF. Consistent with this intake profile, T2 shifted toward a higher-ADG region on the performance map in the late feeding period (Figure 3B), suggesting that once calves adapted from early digestive disturbances, the additional dry matter derived from the 15% MR supported body weight gain during the late feeding period. However, when all periods were integrated, higher MR-derived intake did not translate into a commensurate increase in growth, resulting in a less desirable whole-period FCR than the better-performing strategies, particularly T3 and T4 (Figure 3C; Supplement 3). Recently, a study exploring optimal MR conditions in Hanwoo calves reported results that differ from our observations: the maximum MR DMI was achieved at 20% body weight (v/bw)×20% MR solids, whereas the maximum ADG occurred at 30% body weight (v/bw)×15% MR solids. The same study reported reduced growth and increased diarrhea only when MR solids exceeded 25% [15]. However, these findings contrast with the prevailing interpretation regarding the relationship between MR solids concentration and osmolality. Based on evidence across multiple studies, Azevedo et al. [16] noted that MR osmolality exceeding approximately 600 mOsm/kg can predispose calves to osmotic diarrhea, and that this range may be reached at total solids concentrations of approximately 16%–18% under common MR formulations, particularly with high-volume feeding.

To evaluate the effect of extending the early feeding period, its duration was increased from 30 d to 37 d in T3 while maintaining the same MR concentration and feeding frequency as the control (Table 2, Figure 1). As expected, because the early feeding period itself was 7 d longer in T3 than in the other treatments, cumulative MR and SF DMI during the early feeding period were greater in T3 (Figures 2A, 2B). A distinctive feature of T3 was its position on the early-feeding-period performance map (Figure 3A). Unlike the other treatments, T3 was clearly separated toward the higher intake and higher ADG region and showed the most favorable FCR. This separation should be interpreted with the recognition that the early feeding period metrics in T3 were calculated over 37 d rather than 30 d, and calves undergo rapid growth during this interval. Nevertheless, the pronounced displacement of T3 suggests that prolonging MR-centered feeding during a period of high growth potential contributed to enhanced early growth and improved the efficiency of converting total DMI into body weight gain. In the late feeding period, T3 shifted toward a lower-intake distribution and exhibited lower ADG with poorer FCR, indicating that the efficiency advantage observed earlier was attenuated once MR allowance began to decline (Figure 3B). It is possible that extended exposure to a higher MR supply reduced the urgency for calves to increase SF intake during the late feeding period, such that SF intake did not increase enough to sustain the early efficiency advantage. Nevertheless, when all periods were integrated, extending the MR-feeding in early period improved overall growth efficiency greatly (Figure 3C; Supplement 3).

A previous report suggested that applying a SD reduction of MR during the weaning transition can enhance SF intake and growth efficiency compared with linear tapering in Holstein calves [1]. To evaluate this concept under the present conditions with Hanwoo calves, an SD strategy was implemented in T4 during the late feeding period (Table 2, Figure 1). In T4, MR allowance was reduced by 0.8 L/d for the first 5 d of the late feeding period, maintained at 4 L/d for the subsequent 20 d, and then reduced again by 0.8 L/d during the final 5 d before weaning. This SD design aimed to stimulate calves’ motivation to consume SF by imposing a relatively sharper reduction in MR, thereby encouraging SF intake during the weaning transition. Regarding intake, MR DMI in both the early and late feeding periods in T4 remained broadly comparable to the control (Figure 2A), whereas SF DMI in the late feeding period tended to be the highest among treatments (Figures 2B, 2C). T4 fell within the main distribution with other groups on the performance map (Figure 3A), indicating no distinctive advantage in growth efficiency when nutrient supply was still largely MR-driven. In contrast, during the late feeding period, T4 shifted toward a region characterized by higher ADG at a comparable intake range with lower FCR, consistent with increased SF intake under the SD schedule (Figure 3B). When data across periods were pooled, this late period advantage from the increased SF intake translated into a strong overall growth performance profile for T4 (Figure 3C), supporting previous reports that SD strategies can improve growth efficiency by promoting SF intake, particularly during the weaning transition period [1]. A plausible explanation is that the relatively sharper initial reduction in MR may increase hunger-driven motivation, thereby stimulating greater SF intake, while maintaining MR at 4 L/d for 20 d provided a nutritional buffer that helped avoid an abrupt nutrient shortfall as calves adapted to higher SF consumption. Overall, compared with other feeding programs, the SD-based T4 regimen achieved the higher growth performance, including better ADG, FCR, and weaning body weight, accompanied by higher SF intake (Figures 2, 3; Supplement 3).

**Figure 4:**
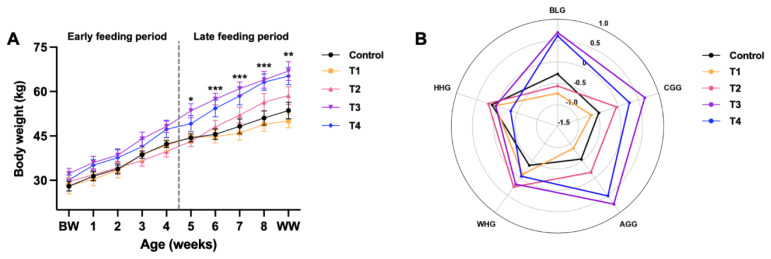
Body weight trajectories and body size growth patterns of Hanwoo calves during milk replacer feeding programs. (A) Weekly body weight trajectories from birth to weaning. Asterisks indicate significant differences among groups (* p<0.05; ** p<0.01; *** p<0.001). (B) Radar plot of standardized body size gains from week 1 to weaning across treatments. Values were standardized as z-scores, (gain value of each index – overall mean across treatments)/SD, to enable comparisons across indices. A z-score of 0 indicates the overall mean, with positive and negative values indicating above- and below-average gains, respectively. BLG, body length gain; CGG, chest girth gain; AGG, abdominal girth gain; WHG, withers height gain; HHG, hip height gain; SD, standard deviation; T1, feeding frequency and meal size; T2, MR solids concentration; T3, early feeding period duration; T4, tapering strategy from MR to SF.

Body weight trajectories provided additional evidence supporting the treatment-specific growth performance patterns described above (Figure 4A; Supplement 3). Although birth weight did not differ among groups, clear separation began to emerge from approximately week 5, suggesting that the cumulative effects of the feeding strategies became most evident during the late feeding period. Relative to the control, T1 showed persistently lower weight accretion toward weaning, consistent with its failure to increase SF intake during the late feeding period under the high-frequency MR schedule. In T2, early growth appeared transiently constrained under the higher-solids MR regimen, whereas growth partially rebounded as calves stabilized during the late feeding period; however, this rebound did not fully offset the earlier growth depression. In contrast, T3 maintained a higher and more sustained growth trajectory through weaning, consistent with the extended MR provision during the early feeding period, whereas T4 showed enhanced late-period growth in association with greater SF intake induced by the SD MR reduction strategy.

Body size growth patterns further supported these interpretations (Figure 4B; Supplement 4). To compare structural development among treatments while accounting for multiple morphometric indices, gains from week 1 (the first morphometric measurement) to weaning were calculated for body length gain (BLG), chest girth gain (CGG), abdominal girth gain (AGG), withers height gain (WHG), and hip height gain (HHG). These gains were standardized as z-scores to generate body size growth profiles for each treatment. Overall, the standardized profiles closely mirrored the patterns observed for growth performance and body weight trajectories, indicating that T3 and T4 promoted not only greater weight accretion but also enhanced structural development. Notably, T3 exhibited relatively balanced gains across all indices compared with the other treatments. In both T3 and T4, the standardized gains for CGG, AGG, and BLG were generally more pronounced than those for WHG and HHG, suggesting that the feeding programs influenced thoracic and abdominal development and longitudinal growth more detectably than skeletal height within the experimental period. Because chest girth is widely used as a practical morphometric proxy for estimating body weight in growing calves and heifers, the greater CGG observed in T3 and T4 provides a biologically meaningful structural correlate to their superior growth outcomes.

Meanwhile, because mean birth weight and sex ratio were not identical across treatments despite no statistically significant difference in birth weight among groups, potential confounding in treatment-specific comparisons was evaluated. Accordingly, sex was included as a fixed effect to adjust for potential confounding due to the unequal sex distribution among treatment groups, rather than to interpret sex-related biological differences. Although mean birth weights were numerically higher in T3 and T4, the statistically significant divergence in body weight became clearly apparent only after approximately week 6, supporting a treatment-driven accumulation of differences during the feeding program rather than a baseline-driven artifact. This approach is supported by previous studies showing absent or inconsistent sex effects on growth performance in pre-weaning calves under controlled milk or MR feeding conditions [17,18]. Overall, the observed changes in feed intake, growth performance, and body-size parameters in the present study were more closely associated with treatment effects than with sex-related effects.

**Figure 5:**
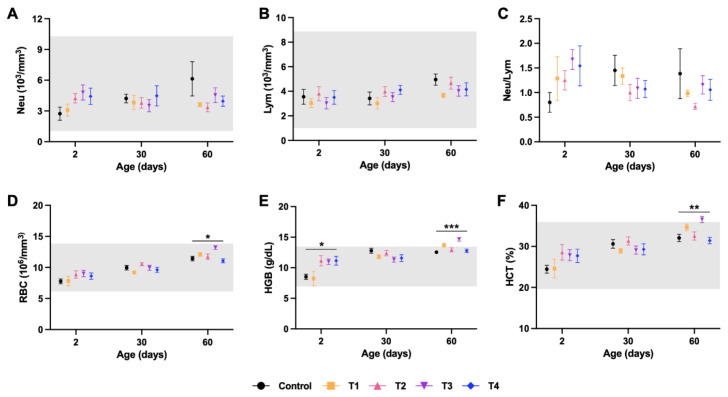
Complete blood count (CBC) parameters of Hanwoo calves according to milk replacer feeding programs. (A) Neutrophil (Neu), (B) lymphocyte (Lym), (C) neutrophil-to-lymphocyte ratio (Neu/Lym), (D) red blood cell (RBC), (E) hemoglobin (HGB), and (F) hematocrit (HCT) parameters of Hanwoo calves according to milk replacer feeding programs at 2, 30, and 60 d of age. Values are presented as means±SEM. Asterisks indicate significant differences among groups at each age (* p<0.05, ** p<0.01, *** p<0.001). Shaded areas indicate reference ranges cited in the text; graphs without shading indicate parameters with no identifiable reference ranges in previous reports. SEM, standard error of the mean; T1, feeding frequency and meal size; T2, MR solids concentration; T3, early feeding period duration; T4, tapering strategy from MR to SF.

CBC and SBP data were evaluated at 2 d, 30 d, and 60 d (Supplements 1, 2), with reference intervals for Hanwoo calves adopted from Kim et al. [19] for CBC and Lee et al. [20] for SBP. In the CBC profile (Figure 5), neutrophils (Neu; 1.9–10.2×10^3^/mm^3^) and lymphocytes (Lym; 0.9–8.9×10^3^/mm^3^) were largely within reference ranges (Figures 5A, 5B), and the Neu/Lym ratio generally declined with age, consistent with expected postnatal immune maturation in calves [19,21]. Notably, the control group showed an apparent outlying individual in Neu/Lym, indicating that appreciable between-calf variability existed even under the same feeding program (Figure 5C). Erythrocyte indices; red blood cell count (RBC; 6.8–14.6×10^6^/mm^3^), hemoglobin (HGB; 6.5–13.5 g/dL), and hematocrit (HCT; 20.4%–39.7%), increased with age across treatments (Figures 5D–5F), reflecting normal hematopoietic development in growing calves [19].

**Figure 6:**
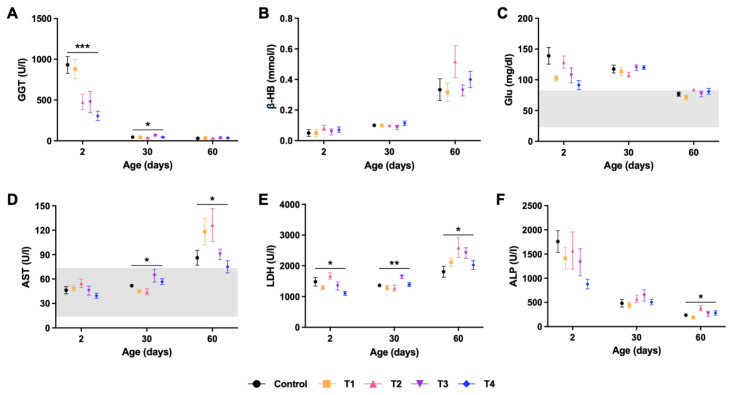
Serum biochemical profiles (SBP) parameters of Hanwoo calves according to milk replacer feeding programs. (A) γ-glutamyl transferase (GGT), (B) β-Hydroxybutyrate (β-HB), (C) glucose (Glu), (D) aspartate aminotransferase (AST), (E) lactate dehydrogenase (LDH), (F) alkaline phosphatase (ALP) parameters of Hanwoo calves according to milk replacer feeding programs at 2, 30, and 60 d of age. Values are presented as means±SEM. Asterisks indicate significant differences among groups at each age (* p<0.05, ** p<0.01, *** p<0.001). Shaded areas indicate reference ranges cited in the text; graphs without shading indicate parameters with no identifiable reference ranges in previous reports. SEM, standard error of the mean; T1, feeding frequency and meal size; T2, MR solids concentration; T3, early feeding period duration; T4, tapering strategy from MR to SF.

Serum γ-glutamyl transferase (GGT) declined sharply after 2 d (Figure 6A). Because GGT is a colostrum-derived enzyme used as an indirect marker of passive transfer, the comparatively modest early GGT response, relative to levels often reported in dairy calves receiving high-volume and high-quality maternal colostrum [22], is consistent with the possibility that commercial colostrum products may provide less effective passive immune transfer than dam-derived colostrum [6]. In this context, the high background incidence of diarrhea observed across groups after the first week in early feeding period may plausibly reflect suboptimal passive transfer under the commercial-colostrum regimen. Nevertheless, because T2 exhibited a distinctly higher diarrhea burden than T3 and T4 despite similarly low early GGT profiles, the excess diarrhea in T2 is more consistent with treatment-specific nutritional factors, particularly the higher MR solids concentration, than with passive transfer alone.

Age-related changes in β-hydroxybutyrate (β-HB) and glucose further supported largely normal metabolic adaptation across programs. Although reference ranges for β-HB were unavailable, β-HB increased progressively with age, whereas plasma glucose (24.85–81.85 mg/dL) declined toward 60 d (Figures 6B, 6C). This coordinated shift is consistent with rumen maturation, whereby energy metabolism relies increasingly on volatile fatty acids produced via ruminal fermentation and less on circulating glucose [23].

Enzymatic markers also provided supportive evidence for a greater metabolic burden in T2. Aspartate aminotransferase (AST; 19.5–72.5 U/L) increased toward weaning in all groups, but T2 showed a pronounced elevation at 60 d that exceeded the reference interval, together with comparatively higher lactate dehydrogenase (LDH) (Figures 6D, 6E). Physiologically, AST is present in both liver and skeletal muscle, and elevations around weaning can reflect increased metabolic demand and tissue turnover [21]. However, the magnitude and treatment specificity in T2 are consistent with an added physiological load under the higher-solids regimen. LDH, a ubiquitous cytosolic enzyme that can rise with heightened tissue metabolism or cellular stress, suggests an added physiological load under the higher-solids regimen [24]. Serum alkaline phosphatase (ALP; reference range unavailable) declined with age across groups (Figure 6F), consistent with the typical developmental trajectory in young calves given its association with bone growth and remodeling [25]. Taken together, these enzyme patterns indicate that although normal developmental changes were observed across all the groups, the AST and LDH response in T2 may be consistent with greater metabolic strain that did not translate into a proportional improvement in growth efficiency under the present conditions [26].

## CONCLUSION

The growth performance and feed efficiency of Hanwoo calves were more strongly influenced by the feeding strategy and the weaning transition process than by total nutrient intake alone. Increasing feeding frequency or MR concentration did not improve productivity and, in some cases, was associated with reduced intake efficiency or increased digestive and metabolic burden. In contrast, extending the early MR feeding period (T3) improved early growth and feed efficiency by maintaining a MR–centered nutrient supply, whereas the SD weaning strategy (T4) promoted a smoother transition to SF by increasing SF intake without compromising growth performance or causing an evident physiological burden.

These findings suggest that successful weaning in Hanwoo calves requires an appropriately designed feeding strategy and weaning transition process matched to the growth stage, rather than a simple increase in nutrient supply. In particular, the SD strategy may have high practical applicability under farm conditions because it can maintain growth performance while reducing feed costs and promoting earlier adaptation to SF. Overall, this study provides evidence for designing a MR feeding program suitable for Hanwoo calves and may contribute to the establishment of an efficient and systematic calf management system. However, the conclusions of this study are limited to the conditions evaluated in the present experiment, and further validation under diverse rearing environments and feeding systems is required. Future studies should also include long-term evaluations of post-weaning growth performance, health status, and economic efficiency.

## Figures and Tables

**Table 1:** Nutritional compositions of the experimental diets

Nutrient composition	Commercial colostrum	Milk replacer	Calf starter	Oat forage
DM (%)	90.2	95.0	91.8	90.0
CF (% of DM)	1.65	0.59	-	-
NDF (% of DM)	-	-	22.0	56.0
ADF (% of DM)	-	-	10.9	34.0
EE (% of DM)	14.9	18.3	5.90	2.5
CP (% of DM)	56.6^1)^	23.5	24.9	9.5
CA (% of DM)	5.61	7.62	7.50	7.2
Lactose (% of DM)	10.8	39.4	-	-
Total sugar (% of DM)	40.0	55.3	-	-

Values for commercial colostrum and milk replacer were obtained by commissioning compositional analyses from the Korea Feed Ingredients Association. Values for calf starter and oat forage were based on manufacturer specifications.

1) Approximately 45% of CP is bovine IgG, according to the manufacturer’s information.

DM, dry matter; CF, crude fiber; NDF, neutral detergent fiber; ADF, acid detergent fiber; EE, ether extract; CP, crude protein; CA, crude ash.

**Table 2:** Experimental conditions of milk replacer feeding programs

Experimental groups	Experimental conditions of milk replacer feeding programs				

Experimental groups	Total milk replacer allowance (L/d)	Feed volume per meal and frequency (L×times/d)	Milk replacer concentration (%)^1)^	Duration of early feeding period (d)	Tapering method during late feeding period (LD^2)^ or SD^3)^)
Control (n = 6: 2F, 4M)	8	2.0×4	13	30	LD
Test 1 (n = 6: 2F, 4M)	8	1.5×6^4)^	13	30	LD
Test 2 (n = 6: 3F, 3M)	8	2.0×4	15	30	LD
Test 3 (n = 7: 4F, 3M)	8	2.0×4	13	37	LD
Test 4 (n = 7: 1F, 6M)	8	2.0×4	13	30	SD

1) Nukamel Yellow at 13% contained 3.03% crude protein, 2.37% crude fiber, and 7.29% total sugar; at 15%, the corresponding values were 3.50%, 2.74%, and 8.29%, respectively.

2) Linear-decrease (LD): In the late feeding period, Milk replacer was gradually reduced by 266 mL/d in the control, T1, and T2 groups until weaning, and by 348 mL/d in the T3 group until weaning.

3) Step-down (SD): Milk replacer allowance was gradually reduced by 800 mL/d for the first 5 days of the late feeding period, maintained at 4 L/d for the following 20 d, and again gradually reduced by 800 mL/d during the last 5 d before weaning.

4) The feeding program consisted of six feedings of 1.5 L each, with a preset maximum daily allowance of 8 L; provision of milk replacer was stopped upon reaching this limit.

## Data Availability

Upon reasonable request, the datasets of this study can be available from the corresponding author.

## SUPPLEMENTARY MATERIAL

**Supplement 1.** Hematological profiles of Hanwoo calves with different milk replacer feeding programs.

**Supplement 2.** Serum biochemical profiles of Hanwoo calves with different milk replacer feeding programs.

**Supplement 3.** Feed intake and growth performance of Hanwoo calves with different milk replacer feeding programs.

**Supplement 4.** Body size profiles of Hanwoo calves with different milk replacer feeding programs.
