# Supplementary material for: Evaluation of weaning strategies using an automated milk replacer feeder on growth performance and physiological responses in Hanwoo calves
Source: Anim Biosci. 2026 Jun 1;39(8):260116. doi: 10.5713/ab.260116 (PMC13441316; doi:10.5713/ab.260116)
Supplement: Supplementary file 1 [file ab-260116-Supplement-1.pdf]

Supplement 1. Hematological profiles of Hanwoo calves with different milk replacer feeding programs

Parameters[1)]	Mean±SEM[2)]					*P* value[3)]		
	Control n=6 (M 2, F 4)	T1 n=6 (M 2, F 4)	T2 n=6 (M 3, F 3)	T3 n=7 (M 4, F 3)	T4 n=7 (M 6, F 1)	Group	Sex	Group×Sex
WBC (10^3/mm^3)								
2 d	7.23±1.21	6.97±0.49	9.07±0.82	8.73±1.21	8.76±1.11	0.569	0.514	0.430
30 d	8.73±0.59	9.10±1.55	8.37±0.69	7.71±0.79	9.16±1.39	0.985	0.083	0.914
60 d	11.80±1.70	7.75±0.28	9.07±0.99	9.43±1.12	8.83±0.90	0.057	0.755	0.194
Neu (10^3/mm^3)								
2 d	2.73±0.64	3.08±0.60	4.23±0.45	4.80±0.74	4.43±0.80	0.272	0.828	0.275
30 d	4.22±0.42	3.82±0.70	3.78±0.51	3.51±0.60	4.47±0.98	0.991	0.237	0.925
60 d	6.13±1.67	3.62±0.20	3.35±0.44	4.54±0.72	3.96±0.50	0.065	0.649	0.271
Lym (10^3/mm^3)								
2 d	3.55±0.61	3.03±0.37	3.80±0.58	3.03±0.46	3.50±0.55	0.799	0.570	0.845
30 d	3.42±0.53	3.02±0.45	3.98±0.39	3.53±0.36	4.11±0.37	0.678	0.460	0.799
60 d	4.95±0.45	3.67±0.17	4.67±0.49	4.03±0.43	4.16±0.53	0.329	0.593	0.216
Neu/Lym								
2 d	0.80±0.20	1.28±0.45	1.25±0.20	1.67±0.20	1.54±0.41	0.428	0.696	0.185
30 d	1.45±0.31	1.33±0.17	1.00±0.16	1.09±0.20	1.07±0.17	0.682	0.381	0.879
60 d	1.38±0.51	0.98±0.07	0.72±0.07	1.16±0.19	1.06±0.21	0.239	0.419	0.289
RBC (10^6/mm^3)								
2 d	7.75±0.39	7.80±0.74	8.87±0.59	9.01±0.50	8.60±0.50	0.483	0.605	0.983
30 d	9.95±0.37	9.18±0.22	10.52±0.23	9.94±0.40	9.61±0.40	0.167	0.653	0.328
60 d	11.4±0.35[b]	12.1±0.29[ab]	11.7±0.45[ab]	13.2±0.31[a]	11.1±0.32[b]	0.011	0.547	0.961

HGB (g/dL)								
2 d	8.52±0.41	8.22±1.18	11.1±0.84	11.00±0.46	11.1±0.71	0.035[4)]	0.808	0.827
30 d	12.8±0.40	11.8±0.30	12.4±0.44	11.3±0.41	11.6±0.56	0.294	0.957	0.534
60 d	12.5±0.27[b]	13.7±0.29[ab]	12.9±0.34[b]	14.7±0.31[a]	12.8±0.29[b]	0.001	0.834	0.428
HCT (%)								
2 d	24.5±0.96	24.6±2.28	28.6±1.91	27.9±1.33	27.7±1.65	0.396	0.851	0.974
30 d	30.6±1.06	28.9±0.57	31.3±0.98	29.1±0.10	29.3±1.35	0.381	0.452	0.245
60 d	32.1±0.88[b]	34.7±0.79[ab]	32.5±1.01[b]	36.6±0.76[a]	31.4±0.78[b]	0.006	0.486	0.977
PLT ($10^3/mm^3$)								
2 d	427.3±37.8	485.0±65.4	441.5±77.8	615.0±90.7	612.0±111.4	0.540	0.391	0.219
30 d	523.5±28.4	497.2±22.6	489.8±36.7	471.4±28.5	496.7±45.2	0.941	0.341	0.811
60 d	406.3±64.9	560.7±43.1	534.0±44.2	527.4±60.7	608.6±36.5	0.115	0.301	0.224

[1)] WBC, white blood cell count; Neu, neutrophil count; Lym, lymphocyte count; Neu/Lym, neutrophil-to-lymphocyte ratio; RBC, red blood cell count; HGB, hemoglobin; HCT, hematocrit; PLT, platelet count. Measurements were performed at 2, 30, and 60 days of age.

[2)] All the values are presented as mean ± SEM (Standard Error of the Mean)

[3)] Group, effect of treatment group; Sex, effect of sex; Group × Sex, interaction effect between group and sex.

[4)] A significant overall group effect was detected; however, none of the pairwise comparisons remained significant after Tukey–Kramer adjustment for multiple comparisons (adjusted P > 0.05).
