# Supplementary material for: Evaluation of weaning strategies using an automated milk replacer feeder on growth performance and physiological responses in Hanwoo calves
Source: Anim Biosci. 2026 Jun 1;39(8):260116. doi: 10.5713/ab.260116 (PMC13441316; doi:10.5713/ab.260116)
Supplement: Supplementary file 2 [file ab-260116-Supplement-2.pdf]

**Supplement 2. Serum biochemical profiles of Hanwoo calves with different milk replacer feeding programs**

| Parameters[1)] | Mean±SEM[2)] | | | | | *P* value[3)] | | |
|---|---|---|---|---|---|---|---|---|
| | Control<br>n=6 (M 2, F 4) | T1<br>n=6 (M 2, F 4) | T2<br>n=6 (M 3, F 3) | T3<br>n=7 (M 4, F 3) | T4<br>n=7 (M 1, F 6) | Group | Sex | Group×Sex |
| TP (g/dL) | | | | | | | | |
| 2 d | 6.60±0.39 | 6.92±0.20 | 6.57±0.38 | 6.53±0.67 | 6.14±0.40 | 0.937 | 0.906 | 0.844 |
| 30 d | 5.80±0.20 | 5.85±0.29 | 5.67±0.29 | 6.40±0.14 | 6.17±0.23 | 0.053 | 0.047 | 0.492 |
| 60 d | 6.07±0.20 | 7.10±0.35 | 7.08±0.19 | 6.81±0.25 | 6.60±0.28 | 0.019[4)] | 0.025 | 0.459 |
| Alb (g/dL) | | | | | | | | |
| 2 d | 2.96±0.10 | 2.90±0.09 | 3.00±0.13 | 3.05±0.12 | 2.77±0.12 | 0.671 | 0.667 | 0.570 |
| 30 d | 3.17±0.12 | 3.13±0.12 | 2.98±0.15 | 3.24±0.05 | 3.21±0.07 | 0.179 | 0.157 | 0.199 |
| 60 d | 2.85±0.06[b] | 3.05±0.08[ab] | 3.15±0.10[ab] | 3.20±0.08[a] | 3.14±0.11[ab] | 0.017 | 0.014 | 0.079 |
| Glb (g/dL) | | | | | | | | |
| 2 d | 3.75±0.26 | 4.02±0.17 | 3.57±0.28 | 3.67±0.48 | 3.37±0.30 | 0.839 | 0.666 | 0.647 |
| 30 d | 2.63±0.08 | 2.72±0.19 | 2.68±0.15 | 3.16±0.12 | 2.96±0.19 | 0.039[4)] | 0.042 | 0.753 |
| 60 d | 3.22±0.15 | 4.05±0.37 | 3.93±0.19 | 3.61±0.22 | 3.46±0.25 | 0.142 | 0.138 | 0.698 |
| T-Chol (mg/dL) | | | | | | | | |
| 2 d | 69.8±5.61 | 72.2±3.66 | 66.5±6.42 | 72.7±7.99 | 53.9±4.78 | 0.793 | 0.232 | 0.747 |
| 30 d | 111.8±6.77 | 102.5±7.99 | 91.3±9.43 | 106.6±5.90 | 107.4±4.78 | 0.211 | 0.093 | 0.114 |
| 60 d | 123.3±11.41 | 112.7±7.06 | 103.0±4.44 | 111.9±6.79 | 104.0±5.45 | 0.299 | 0.544 | 0.398 |
| TG (mg/dL) | | | | | | | | |
| 2 d | 49.5±10.54 | 46.7±11.88 | 13.7±1.59 | 19.4±4.42 | 15.4±2.06 | 0.016[4)] | 0.580 | 0.684 |
| 30 d | 14.3±0.72[ab] | 18.2±2.15[ab] | 11.0±0.68[b] | 21.6±2.98[a] | 18.7±2.71[ab] | 0.043 | 0.164 | 0.894 |

| | | | | | | | | |
|---|---|---|---|---|---|---|---|---|
| 60 d | 12.7±1.17$^{b}$ | 11.3±0.88$^{b}$ | 12.8±0.70$^{ab}$ | 17.1±1.35$^{a}$ | 13.7±1.23$^{ab}$ | 0.024 | 0.799 | 0.497 |
| LDL (mg/dL) | | | | | | | | |
| 2 d | 25.0±1.51 | 25.7±1.28 | 27.3±3.28 | 33.7±3.69 | 25.4±2.07 | 0.206 | 0.080 | 0.576 |
| 30 d | 44.0±2.25 | 43.2±4.57 | 45.3±4.32 | 45.9±2.60 | 48.6±2.50 | 0.237 | 0.169 | 0.048 |
| 60 d | 64.5±5.67 | 62.8±3.30 | 54.2±3.09 | 59.1±5.03 | 53.9±3.92 | 0.192 | 0.733 | 0.340 |
| HDL (mg/dL) | | | | | | | | |
| 2 d | 45.0±4.10 | 47.3±2.81 | 43.3±4.67 | 48.7±5.93 | 35.9±3.97 | 0.879 | 0.173 | 0.523 |
| 30 d | 65.3±4.49 | 59.8±3.50 | 57.5±5.64 | 70.3±4.01 | 70.7±2.93 | 0.045[4)] | 0.249 | 0.258 |
| 60 d | 74.3±6.76 | 70.0±4.28 | 65.5±2.63 | 67.9±2.92 | 63.9±3.26 | 0.248 | 0.377 | 0.112 |
| LDL/HDL | | | | | | | | |
| 2 d | 0.58±0.07 | 0.53±0.02 | 0.63±0.05 | 0.71±0.04 | 0.73±0.04 | 0.105 | 0.683 | 0.140 |
| 30 d | 0.67±0.03$^{ab}$ | 0.73±0.05$^{ab}$ | 0.78±0.02$^{a}$ | 0.64±0.02$^{b}$ | 0.69±0.04$^{ab}$ | 0.042 | 0.872 | 0.049 |
| 60 d | 0.87±0.04 | 0.90±0.03 | 0.83±0.05 | 0.86±0.04 | 0.86±0.02 | 0.764 | 0.385 | 0.226 |
| GGT (IU/L) | | | | | | | | |
| 2 d | 931.8±102.6$^{a}$ | 882.0±119.8$^{ab}$ | 477.2±95.7$^{bc}$ | 474.3±129.8$^{bc}$ | 305.6±58.1$^{c}$ | 0.001 | 0.975 | 0.422 |
| 30 d | 46.2±5.44$^{ab}$ | 42.7±4.51$^{ab}$ | 38.2±4.42$^{b}$ | 66.0±7.29$^{a}$ | 44.9±5.99$^{ab}$ | 0.029 | 0.382 | 0.987 |
| 60 d | 30.0±4.49 | 35.2±3.44 | 31.3±4.01 | 33.1±4.01 | 35.6±6.17 | 0.938 | 0.859 | 0.553 |
| NEFA (mmol/L) | | | | | | | | |
| 2 d | 0.15±0.03 | 0.13±0.02 | 0.12±0.02 | 0.20±0.03 | 0.17±0.03 | 0.115 | 0.122 | 0.329 |
| 30 d | 0.18±0.02 | 0.16±0.02 | 0.17±0.02 | 0.24±0.02 | 0.24±0.04 | 0.165 | 0.238 | 0.820 |
| 60 d | 0.17±0.03 | 0.15±0.02 | 0.17±0.02 | 0.19±0.03 | 0.20±0.02 | 0.372 | 0.807 | 0.280 |
| BUN (mg/dL) | | | | | | | | |
| 2 d | 5.33±0.422$^{b}$ | 6.17±0.87$^{ab}$ | 8.83±0.83$^{ab}$ | 7.43±0.99$^{ab}$ | 9.33±1.20$^{a}$ | 0.012 | 0.122 | 0.531 |
| 30 d | 8.50±0.428 | 8.17±0.87 | 9.00±0.58 | 7.71±0.78 | 7.86±0.63 | 0.486 | 0.958 | 0.070 |

| | | | | | | | | |
|---|---|---|---|---|---|---|---|---|
| 60 d | 11.7±1.453$^{ab}$ | 15.5±0.72$^{a}$ | 11.0±0.45$^{b}$ | 11.4±0.37$^{b}$ | 11.6±1.11$^{ab}$ | 0.018 | 0.272 | 0.425 |
| b-HB (mmol/L) | | | | | | | | |
| 2 d | 0.05±0.02 | 0.05±0.02 | 0.08±0.02 | 0.06±0.02 | 0.07±0.02 | 0.741 | 0.857 | 0.059 |
| 30 d | 0.10±0.00 | 0.10±0.00 | 0.10±0.00 | 0.09±0.02 | 0.11±0.02 | 0.609 | 0.750 | 0.609 |
| 60 d | 0.33±0.07 | 0.32±0.06 | 0.52±0.11 | 0.33±0.04 | 0.40±0.06 | 0.298 | 0.662 | 0.437 |
| Glu (mg/dL) | | | | | | | | |
| 2 d | 139.0±13.6 | 102.2±4.49 | 128.8±10.0 | 107.3±12.2 | 91.4±7.35 | 0.183 | 0.978 | 0.820 |
| 30 d | 117.5±6.46 | 113.5±6.81 | 108.3±4.03 | 119.6±4.48 | 119.9±3.03 | 0.397 | 0.520 | 0.499 |
| 60 d | 76.7±3.42 | 71.2±4.11 | 84.2±2.70 | 77.1±4.75 | 81.3±4.72 | 0.235 | 0.029 | 0.192 |
| T-Bil (μmol/L) | | | | | | | | |
| 2 d | 0.44±0.08 | 0.48±0.11 | 0.33±0.05 | 0.32±0.06 | 0.31±0.10 | 0.413 | 0.732 | 0.523 |
| 30 d | 0.22±0.03 | 0.23±0.03 | 0.15±0.02 | 0.23±0.02 | 0.19±0.04 | 0.269 | 0.642 | 0.638 |
| 60 d | 0.10±0.00 | 0.10±0.00 | 0.10±0.00 | 0.13±0.03 | 0.10±0.00 | 0.676 | 0.501 | 0.676 |
| D-Bil (μmol/L) | | | | | | | | |
| 2 d | 0.23±0.03 | 0.23±0.05 | 0.18±0.03 | 0.23±0.05 | 0.19±0.04 | 0.731 | 0.813 | 0.201 |
| 30 d | 0.12±0.02 | 0.12±0.02 | 0.10±0.00 | 0.14±0.02 | 0.13±0.02 | 0.419 | 0.560 | 0.418 |
| 60 d | 0.03±0.02 | 0.02±0.02 | 0.08±0.02 | 0.09±0.01 | 0.04±0.02 | 0.057 | 0.424 | 0.492 |
| AST (IU/L) | | | | | | | | |
| 2 d | 46.3±4.39 | 48.3±3.61 | 54.7±5.20 | 45.7±5.56 | 39.4±3.09 | 0.411 | 0.952 | 0.502 |
| 30 d | 51.8±1.68 | 45.0±1.61 | 44.3±3.56 | 64.1±7.87 | 56.7±3.54 | 0.033[4)] | 0.937 | 0.600 |
| 60 d | 86.2±9.16 | 118.2±16.2 | 126.5±20.1 | 90.4±6.51 | 75.0±7.39 | 0.025[4)] | 0.192 | 0.330 |
| Crea (mg/dL) | | | | | | | | |
| 2 d | 1.20±0.08 | 1.23±0.06 | 1.33±0.04 | 1.24±0.10 | 1.25±0.11 | 0.526 | 0.601 | 0.346 |
| 30 d | 1.08±0.06 | 1.05±0.07 | 1.18±0.05 | 1.13±0.05 | 1.17±0.03 | 0.475 | 0.839 | 0.994 |

| | | | | | | | | |
|---|---|---|---|---|---|---|---|---|
| 60 d | 1.22±0.07 | 1.20±0.03 | 1.10±0.03 | 1.10±0.04 | 0.97±0.05 | 0.001[4)] | 0.010 | 0.142 |
| LDH (IU/L) | | | | | | | | |
| 2 d | 1480.5±139.4$^{ab}$ | 1286.5±61.0$^{ab}$ | 1669.7±110.1$^{a}$ | 1339.9±128.1$^{ab}$ | 1104.4±65.3$^{b}$ | 0.035 | 0.384 | 0.877 |
| 30 d | 1360.0±28.9$^{b}$ | 1287.3±75.0$^{b}$ | 1272.7±97.7$^{b}$ | 1645.7±56.7$^{a}$ | 1390.0±62.5$^{ab}$ | 0.002 | 0.830 | 0.168 |
| 60 d | 1804.7±178.7$^{b}$ | 2113.3±139.7$^{ab}$ | 2592.3±318.1$^{a}$ | 2418.9±170.4$^{ab}$ | 2023.6±140.8$^{ab}$ | 0.032 | 0.409 | 0.159 |
| ALP (IU/L) | | | | | | | | |
| 2 d | 1758.5±223.8 | 1412.2±228.4 | 1570.3±386.1 | 1328.0±280.8 | 877.9±99.4 | 0.372 | 0.764 | 0.942 |
| 30 d | 483.3±80.2 | 445.7±56.8 | 574.3±74.1 | 644.4±116.9 | 506.1±57.9 | 0.399 | 0.763 | 0.062 |
| 60 d | 238.7±15.8$^{ab}$ | 189.7±15.9$^{b}$ | 383.5±55.8$^{a}$ | 266.6±50.3$^{ab}$ | 282.4±46.0$^{ab}$ | 0.042 | 0.314 | 0.243 |
| Ca (mg/dL) | | | | | | | | |
| 2 d | 11.9±0.87 | 11.5±0.46 | 10.4±0.70 | 10.6±0.89 | 9.76±0.30 | 0.536 | 0.831 | 0.915 |
| 30 d | 8.93±0.59$^{abc}$ | 8.78±0.26$^{bc}$ | 8.23±0.47$^{c}$ | 10.2±0.29$^{a}$ | 9.83±0.35$^{ab}$ | 0.003 | 0.069 | 0.415 |
| 60 d | 8.47±0.38$^{c}$ | 8.60±0.13$^{abc}$ | 9.42±0.19$^{a}$ | 9.16±0.25$^{ab}$ | 8.90±0.26$^{bc}$ | 0.001 | 0.001 | 0.026 |
| iP (mg/dL) | | | | | | | | |
| 2 d | 7.43±0.40 | 7.13±0.47 | 7.98±0.29 | 8.63±0.78 | 7.89±0.45 | 0.545 | 0.488 | 0.887 |
| 30 d | 8.10±0.41 | 8.13±0.34 | 8.22±0.44 | 9.56±0.36 | 9.40±0.20 | 0.011[4)] | 0.831 | 0.300 |
| 60 d | 7.68±0.55$^{b}$ | 7.48±0.43$^{b}$ | 9.57±0.23$^{a}$ | 8.64±0.35$^{ab}$ | 8.51±0.44$^{ab}$ | 0.012 | 0.282 | 0.165 |

[1)] TP, total protein; Alb, albumin; Glb, globulin; T-Chol, total cholesterol; TG, triglyceride; LDL, low-density lipoprotein; HDL, high-density lipoprotein; LDL/HDL, low-density lipoprotein to high-density lipoprotein ratio; GGT, γ-glutamyl transferase; NEFA, non-esterified fatty acids; BUN, blood urea nitrogen; β-HB, β-hydroxybutyrate; Glu, glucose; T-Bil, total bilirubin; D-Bil, direct bilirubin; AST, aspartate aminotransferase; Crea, creatinine; LDH, lactate dehydrogenase; ALP, alkaline phosphatase; Ca, calcium; iP, inorganic phosphorus. Measurements were performed at 2, 30, and 60 days of age.

[2)] All the values are presented as mean ± SEM (Standard Error of the Mean)

[3)] Group, effect of treatment group; Sex, effect of sex; Group × Sex, interaction effect between group and sex.

[4)] A significant overall group effect was detected; however, none of the pairwise comparisons remained significant after Tukey–Kramer adjustment for multiple comparisons (adjusted $P > 0.05$).
