# Supplementary material for: Evaluation of weaning strategies using an automated milk replacer feeder on growth performance and physiological responses in Hanwoo calves
Source: Anim Biosci. 2026 Jun 1;39(8):260116. doi: 10.5713/ab.260116 (PMC13441316; doi:10.5713/ab.260116)
Supplement: Supplementary file 3 [file ab-260116-Supplement-3.pdf]

Supplement 3. Feed intake and growth performance of Hanwoo calves with different milk replacer feeding programs

Parameters[1)]	Mean±SEM[2)]					*P* value[3)]		
	Control n=6 (M 2, F 4)	T1 n=6 (M 2, F 4)	T2 n=6 (M 3, F 3)	T3[4)] n=7 (M 4, F 3)	T4 n=7 (M 6, F 1)	Group	Sex	Group×Sex
Milk replacer DMI (kg/head)								
Early feeding period DMI	24.0±0.49[c]	24.2±0.67[c]	27.1±0.82[b]	32.3±0.84[a]	24.1±0.70[bc]	<0.001	0.270	0.064
Late feeding period DMI	17.3±0.03[b]	16.4±0.05[c]	20.4±0.39[a]	12.7±0.24[d]	15.7±0.16[c]	<0.001	0.061	0.651
Total MR DMI (A)	41.3±0.52[b]	40.5±0.70[b]	47.4±0.92[a]	44.9±1.01[a]	39.8±0.75[b]	<0.001	0.138	0.153
Solid feeed[5)] DMI (kg/head)								
Early feeding period DMI	0.54±0.10[b]	0.58±0.24[b]	1.30±0.25[ab]	1.84±0.35[a]	1.34±0.22[ab]	0.009	0.390	0.325
Late feeding period DMI	12.7±2.26	10.2±1.10	10.7±1.64	8.97±1.54	16.4±1.64	0.083	0.552	0.670
Total SF DMI (B)	13.2±2.27	10.8±1.33	12.0±1.87	10.8±1.78	17.7±1.71	0.117	0.662	0.604
Total DMI (A+B) (kg/head)	54.5±2.35	51.4±1.45	59.5±2.53	55.7±2.54	57.6±2.15	0.103	0.385	0.351
Average daily gain (ADG)[6)] (kg/day)								
Early feeding period ADG	0.49±0.04[ab]	0.47±0.03[ab]	0.42±0.03[b]	0.63±0.04[a]	0.58±0.06[ab]	0.014	0.769	0.808
Late feeding period ADG	0.36±0.05[ab]	0.26±0.04[b]	0.56±0.06[a]	0.49±0.08[ab]	0.59±0.02[ab]	0.012	0.268	0.714
ADG (all periods)	0.43±0.03[b]	0.37±0.03[b]	0.49±0.04[ab]	0.58±0.04[a]	0.59±0.03[a]	0.001	0.407	0.712
Feed conversion ratio (FCR)[7)]								
Early feeding period FCR	1.71±0.14[ab]	1.82±0.14[ab]	2.33±0.16[a]	1.51±0.13[b]	1.57±0.18[b]	0.011	0.452	0.774
Late feeding period FCR	3.07±0.43	3.72±0.55	1.96±0.20	2.68±0.83	1.82±0.09	0.209	0.360	0.587

FCR (all periods)	2.17±0.11^{a}	2.39±0.16^{a}	2.08±0.10^{ab}	1.63±0.08^{b}	1.67±0.11^{b}	0.001	0.640	0.582
Body weight (kg)								
Birth weight	28.0±1.59	28.1±2.71	29.5±1.41	32.3±1.81	30.06±1.75	0.342	0.670	0.178
1 wk	31.4±1.55	30.4±2.20	31.8±1.80	36.0±2.14	35.11±1.87	0.505	0.264	0.264
2 wk	33.8±1.73	33.3±2.54	34.3±1.90	38.5±2.15	37.66±2.68	0.218	0.457	0.271
3 wk	38.7±1.24	38.8±2.25	36.7±1.84	44.0±2.25	41.40±2.45	0.114	0.539	0.308
4 wk	42.3±1.15	41.8±2.03	39.8±1.93	48.2±2.07	47.23±2.82	0.015[8)]	0.723	0.156
5 wk	44.4±1.34^{ab}	44.6±2.09^{ab}	43.2±1.77^{b}	53.5±2.41^{a}	49.11±2.78^{ab}	0.011	0.802	0.322
6 wk	45.5±1.78^{b}	44.8±2.18^{b}	48.1±2.12^{ab}	57.4±2.00^{a}	54.31±2.87^{a}	<0.001	0.513	0.250
7 wk	48.3±2.48^{b}	45.9±2.30^{b}	52.0±2.66^{ab}	60.9±2.26^{a}	58.51±3.06^{a}	<0.001	0.474	0.352
8 wk	51.1±2.39^{b}	48.9±2.41^{b}	56.4±2.90^{ab}	63.9±2.86^{a}	63.14±2.78^{a}	<0.001	0.317	0.377
Weaning weight	53.6±2.84^{b}	50.1±2.36^{b}	58.6±3.02^{ab}	66.9±3.17^{a}	65.20±2.88^{a}	0.001	0.399	0.269

1) DMI, dry matter intake; MR, milk replacer; SF, solid feed; wk, week of age.

2) All the values are presented as mean ± SEM (Standard Error of the Mean)

3) Group, effect of treatment group; Sex, effect of sex; Group × Sex, interaction effect between group and sex.

4) For T3, the parameters (DMI, ADG, and FCR) were derived from a 37-day early feeding period and a 23-day late feeding period. In contrast, the parameters for the other groups were calculated based on 30 days for each period.

5) Solid feed was provided as a mixture of calf starter and oat forage at a ratio of 9 : 1.

6) ADG was calculated as body weight gain (kg) divided by feeding days (d).

7) FCR was calculated as total DMI (kg) divided by body weight gain (kg).

8) A significant overall group effect was detected; however, none of the pairwise comparisons remained significant after Tukey–Kramer adjustment for multiple comparisons (adjusted $P > 0.05$).
