# Supplementary material for: Evaluation of weaning strategies using an automated milk replacer feeder on growth performance and physiological responses in Hanwoo calves
Source: Anim Biosci. 2026 Jun 1;39(8):260116. doi: 10.5713/ab.260116 (PMC13441316; doi:10.5713/ab.260116)
Supplement: Supplementary file 4 [file ab-260116-Supplement-4.pdf]

Supplement 4. Body size profiles of Hanwoo calves with different milk replacer feeding programs

Parameters[1)]	Mean±SEM[3)]					*P* value[4)]		
	Control n=6 (M 2, F 4)	T1 n=6 (M 2, F 4)	T2 n=6 (M 3, F 3)	T3 n=7 (M 4, F 3)	T4 n=7 (M 6, F 1)	Group	Sex	Group×Sex
Average daily body length gain (cm)								
BL at 1 wk	63.5±0.76	65.2±2.37	64.7±1.43	64.3±2.15	63.4±1.46	0.966	0.631	0.046
BL at weaning	79.7±1.05	79.0±1.61	79.3±1.54	84.0±0.98	84.3±1.81	0.036[5)]	0.112	0.397
BLG[2)]	16.2±0.95[ab]	13.8±1.28[b]	14.7±1.26[b]	19.7±1.34[ab]	20.9±1.98[a]	0.034	0.305	0.593
Average daily chest girth gain (cm)								
CG at 1 wk	73.0±1.15	71.8±1.78	73.3±1.38	75.0±1.35	75.4±1.34	0.097	0.634	0.062
CG at weaning	89.2±1.72[ab]	88.0±0.68[b]	91.7±2.09[ab]	95.7±1.49[a]	94.4±1.19[a]	0.003	0.617	0.054
CGG[2)]	16.2±1.28	16.2±1.30	18.3±2.01	20.7±0.61	19.0±0.87	0.193	0.959	0.636
Average daily abdominal girth gain (cm)								
AG at 1 wk	74.2±1.70	75.0±1.75	76.5±1.43	77.0±1.51	78.7±1.52	0.087	0.861	0.123
AG at weaning	94.5±1.82[bc]	93.0±1.51[c]	98.5±2.69[abc]	103.0±2.36[ab]	105.0±2.72[a]	0.003	0.736	0.139
AGG[2)]	20.3±1.93[ab]	18.0±2.08[b]	22.0±1.90[ab]	26.0±1.11[a]	26.3±1.96[a]	0.011	0.605	0.866
Average daily withers height gain (cm)								
WH at 1 wk	72.2±0.95	71.3±0.49	69.8±1.56	74.1±1.34	73.1±0.99	0.110	0.731	0.192
WH at weaning	82.8±0.95	83.5±2.16	83.2±2.21	86.1±0.51	85.6±0.95	0.199	0.216	0.072
WHG[2)]	10.7±1.33	12.2±1.85	13.3±1.82	12.0±1.07	12.4±1.04	0.813	0.139	0.222
Average daily hip height gain (cm)								

HH at 1 wk	72.7±1.12	74.3±2.03	73.2±2.18	77.7±1.74	77.1±0.99	0.123	0.238	0.133
HH at weaning	86.2±0.87	87.8±2.34	87.5±1.84	90.7±1.11	89.1±1.39	0.106	0.386	0.039
HHG[2)]	13.5±1.69	13.5±1.59	14.3±1.48	13.0±1.23	12.0±0.98	0.981	0.630	0.257

[1)] BLG, body length gain; CGG, chest girth gain; AGG, abdominal girth gain; WHG, withers height gain; HHG, hip height gain; wk, week of age.

[2)] BLG, CGG, AGG, WHG, and HHG were calculated as the difference between body size measurements at weaning and at 1 week of age.

[3)] All the values are presented as mean ± SEM (Standard Error of the Mean)

[4)] Group, effect of treatment group; Sex, effect of sex; Group × Sex, interaction effect between group and sex.

[5)] A significant overall group effect was detected; however, none of the pairwise comparisons remained significant after Tukey–Kramer adjustment for multiple comparisons (adjusted P > 0.05).
